# Postbiotic-Enabled Targeting of the Host-Microbiota-Pathogen Interface: Hints of Antibiotic Decline?

**DOI:** 10.3390/pharmaceutics12070624

**Published:** 2020-07-04

**Authors:** Matteo Puccetti, Styliani Xiroudaki, Maurizio Ricci, Stefano Giovagnoli

**Affiliations:** Department of Pharmaceutical Sciences, via del Liceo 1, University of Perugia, 06123 Perugia, Italy; matteo.puccetti@gmail.com (M.P.); styliani.xiroudaki@studenti.unipg.it (S.X.); maurizio.ricci@unipg.it (M.R.)

**Keywords:** infectious diseases, 3-indole carboxaldehyde, microbiota, host-microbiota-pathogen interface, antimicrobial resistance, postbiotics, tryptophan metabolites

## Abstract

Mismanagement of bacterial infection therapies has undermined the reliability and efficacy of antibiotic treatments, producing a profound crisis of the antibiotic drug market. It is by now clear that tackling deadly infections demands novel strategies not only based on the mere toxicity of anti-infective compounds. Host-directed therapies have been the first example as novel treatments with alternate success. Nevertheless, recent advances in the human microbiome research have provided evidence that compounds produced by the microbial metabolism, namely postbiotics, can have significant impact on human health. Such compounds target the host-microbe-pathogen interface rescuing biotic and immune unbalances as well as inflammation, thus providing novel therapeutic opportunities. This work discusses critically, through literature review and personal contributions, these novel nonantibiotic treatment strategies for infectious disease management and resistance prevention, which could represent a paradigm change rocking the foundation of current antibiotic therapy tenets.

## 1. Introduction

The emergence of antimicrobial resistance (AMR) is alarming public health systems worldwide. The emergence of antibiotic resistance leads to an increased risk of therapeutic failure, longer hospitalization, relapses, and greater costs for the health care system. The main challenge in the fight against AMR is the poor efficacy of most of the available treatments, which results in limited prevention or control [1]. Therefore, infections, such as pneumonia, tuberculosis, and foodborne disease are becoming harder to treat [2]. AMR infections are associated with increased mortality, morbidity, and lower quality of life related to the need for difficult, lengthy, and expensive treatments. Resistant pathogens demand more aggressive multiple drug regimens with a high risk of adverse effects and, in some cases, ultimate therapy discontinuation. These regimens often require inpatient settings and long and costly hospitalization. The increased failure frequency of antibiotic treatments raises questions on whether current strategies against AMR will succeed or we are relentlessly heading towards a post antibiotic era [3].

Current reports count 700,000 deaths each year in the world caused by AMR infections. In the US alone, 35,000 deaths a year occur from untreatable infections and mortality is estimated to rise to 10 million new cases by 2050 worldwide [4], alarming numbers that pushed the World Health Organization to warn for urgent interventions [5].

The overuse and misuse of antibiotics in health care, agriculture, food industry, and farming, including unnecessary or incorrect prescriptions, poor patient compliance, and rapid bacterial evolution in response to bactericidal selective pressure tools, have contributed to the rising resistance. Such mismanagement along with poor diagnostic tools and depotentiated discovery and development pipelines are major roots to the current crisis [4,6,7]. In addition, concern is growing around emerging viral infections, such as the current COVID-19, that have been linked to an increased risk of antibiotic abuse and AMR [8].

For a more insightful understanding of the worrying antibiotic crisis, we recommend the reader to refer to more focused recent literature [9,10,11,12,13,14].

In light of the above considerations, breakthrough strategies surpassing classical antibiotic mechanisms are therefore needed to overcome AMR.

Antibiotic treatment alters the population structure of the microbiota, with transient and persistent effects on bacterial diversity and population composition. These changes in the microbial community alter resource availability and interspecies communication, with increased chances for pathogen intrusion and colonization resistance loss [15]. Antibiotic treatment can determine selection of antibiotic-resistant populations and transmission of resistance genes in the microbiome [15]. Thus, more selective approaches affecting pathogens while leaving commensals unharmed is gaining ground.

It is by now clear that tackling deadly infections demands novel strategies not only based on the mere toxicity of anti-infective compounds. The arsenal in the hands of microorganisms is by far exceeding the tools available to prevent and defeat bacterial resistance. Moreover, a growing amount of evidence connects host-microbiota dysregulation to human diseases, which warns further against antibiotic abuse and indiscriminate prescriptions [16,17,18,19].

This growing awareness prompts new approaches to fight infections, even owing to a deeper comprehension of the complex host–microbiota relationship and how the preservation of such a delicate balance impacts human health.

## 2. Nonantibiotic Based Therapies

Current alternative approaches to antibiotics encompass a wide range of possible treatment options, including phage therapy [20], novel vaccines [21], peptides [22], antimicrobial metals [23], and biofilm targeting [24,25], to name a few, with limited success [26,27,28].

Other options have stemmed from the evidence that, albeit challenging, AMR could be treated using factors with pleiotropic mechanisms of action that have the ability to interfere with the molecular pathways required by the pathogen for replication and/or persistence, enabling host-mediated responses to infection. These so-called host directed therapies (HDTs), enhancing protective immune signatures, reducing exacerbated inflammation, or balancing immune reactivity at the infection site, represent one of the major alternative approaches against AMR [29,30,31,32].

Some HDTs exploit the repurposing of drugs commonly used in therapy and that target specific pathways [33,34,35,36]. For instance, metformin has been tested in *Mycobacterium tuberculosis* infections [29,37] since, by activating the 5ʹ-adenosine monophosphate-activated protein kinase (MAPK), it interferes with the mitochondrial respiratory chain, inducing the production of reactive oxygen species and macrophage activation. Other repurposed drugs to treat epilepsy [38], peptic ulcers [39], cancer [40], anti-inflammatory drugs [41,42,43,44,45,46], antibodies [47,48,49], and hypoglycemic compounds [50] or sphingosine-1-phosphate receptor agonists [51] have been investigated.

Micronutrients and immune-modulators, like the granulocyte–macrophage colony-stimulating factor and interferon (IFN)-γ, are used to boost immunity in patients with sepsis [52,53,54]. Moreover, antimicrobial peptide inducers, such as 1,25-dihydroxyvitamin D3 and phenylbutyrate [55,56,57,58], and checkpoint inhibitors, such as programmed cell death-1 inhibitors, are currently in the spotlight for the efficacy in unleashing the immune system for the treatment of chronic infections, such as tuberculosis [59] (Table 1).

Being the pathogen not targeted, HTDs avoid selective pressure on the microbe and, hence, the risk of developing resistance to treatment is small, thus opening a new trove of possibilities. 

A recent holistic approach to antibiotic therapy, able to rescue the dysfunctional host–pathogen interaction, can spark novel opportunities for therapeutic innovation. This new approach exploits microbial therapeutics that, rather than damaging the human host and disrupting the microbial ecosystem, increase the fitness of both. This can result in several benefits: (i) a salutary symbiosis, (ii) reduced selection for additional antibiotic resistance genes, and (iii) decreased transmission rates of antibiotic-resistant bacteria by decolonization of patients and the surrounding community [60].

Indeed, combined with the interest in HDTs, advances in the human microbiome research have produced evidence that the biotic balance is a determinant factor in human health. Antibiotics interfere on the microbiome-immune system axis, resulting in immunological disorders [61,62], and also increase host’s susceptibility to pathogens [63,64,65,66]. It is obvious that the overuse of broad-spectrum antibiotics should be rapidly replaced with more specific approaches and complemented with efficient methods to restore the altered microbiome. 

## 3. The Microbiota Relevance in Human Infections

Infectious disease treatments are undergoing a crucial paradigm shift as the intricate interactions between the human microbiota, defined as the community of microorganisms themselves, the immune system, and human pathogens are slowly being untwined.

An increasing amount of evidence strongly supports the crucial role of the human microbiome in human health and disease [67,68,69]. As a result, the microbiota has become an attractive target for potential therapeutics [70].

### 3.1. The Host–Gut Microbiota Interface 

It is today clear that trillions of microbes colonize all districts of the human body. The human microbiota contributes to human health due to a natural symbiosis that develops with the host shortly after birth. This reciprocal supporting function of the microbiota is today virtually recognized in different organs and compartments. The microbiota composition and function differ according to location, host’s factors, and lifestyle, which determine individual specificity [71]. Currently, the most characterized microbiota is the gut microbiota (GM) [72].

A growing body of evidence, collected through advanced sequencing technologies, underpins the relationship between the changes in the GM composition and diseases. The GM functions by supporting the integrity of the mucosal barrier, providing nutrients, and preventing pathogen invasion. Since the microbiota-mucosal immune system interaction is critical for the host’s immune balance, alterations in the GM composition and function can result into several ailments, including diabetes, obesity, asthma, cancer, cystic fibrosis [73], inflammatory bowel disease (IBD) [74,75], chronic liver disease [76], atopic dermatitis (AD) [77], and chronic mucocutaneous candidiasis [78]. Therefore, the GM equilibrium may be crucial for the delicate balance of the whole-body function and health. Such an important role of the GM seems to be attributable, at least in part, to the production of microbiota-derived metabolites.

### 3.2. Antibiotics and the Gut Microbiota

A considerable research effort has been focused on the comprehension of the link between the effect of antibiotics on specific bacterial populations within the GM and the emergence of drug resistance [79,80,81].

By decreasing the diversity of the microbiota, antibiotics can cause outgrowth of certain bacterial populations, which can convert to pathogens themselves or favor the invasion of alien microbes [82]. Such conditions result in a number of serious pathologic disorders, as in the case of *Clostridium difficile* infections, where the toxins released by the microbe damage the epithelium, leading to inflammation and cell death.

The antibiotic induced alteration of the microbiota perturbates the mucosal pattern recognition receptor (PRR) signaling and the secretion of antimicrobial peptides and impairs the development and function of intestinal immune cells. Innate lymphoid cells (ILC3) play a fundamental role, via an Interleukin (IL)-22-dependent pathway, in the blocking bacteria translocation through the intestinal epithelium. By impacting the ILC3 group recruitment and development, antibiotics knock down IL-22 production, an important cytokine for intestinal homeostasis, augmenting host susceptibility to invading pathogens [83]. Likewise, the GM disruption can dysregulate the T-helper (Th)1 to Th2 cell ratio, the differentiation of naive T cells into regulatory T cells, and deplete the Th17 cell population [84]. A dysbiotic microbiota loses its fundamental regulatory role [85] thereby resulting in metabolic, immunological, and developmental disorders as much as susceptibility to infections [86,87,88,89].

### 3.3. Microbiota-Based Therapeutics

It is today accepted that the GM modulation can be useful to manage gastrointestinal disorders. A dysbiotic microbiota can be prevented by supplying microorganisms to restore impaired microbial populations and to enhance the intestine resiliency to infection. In this regard, prebiotics, probiotics, fecal microbiota transplantation (FMT), and bacteriophage therapy are major treatment options (Table 2).

#### 3.3.1. Fecal Microbiota Transplantation 

Opportunistic antibiotic-resistant infections, such as those from *C. difficile*, can be fought by supplying fecal microbes [102,103]. Healthy fecal microbes engage niche competition with the pathogen, suppressing *C. difficile* propagation through the production of growth inhibitors. FMT can indeed limit the transmission of resistance and extend the viability of antibiotics. Albeit FMT could contribute to the eradication of multidrug-resistant bacteria, the risk for local and bloodstream infections has raised safety concerns in immunocompromised patients [104].

#### 3.3.2. Bacteriophages

Bacteriophages represent the major viral components of the GM and contribute to several GM features, such as microbial composition and diversity as well as horizontal gene transfer [105,106,107,108,109]. Bacteriophages directed against specific bacterial strains can be another viable option against AMR [110]. Bacteriophages show efficacy against biofilm-associated infections, such as methicillin-resistant *Staphylococcus aureus* [111] and *P. aeruginosa* infections [112]. In spite of the major advantages over antibiotics, such as host-specificity [113], phage therapy, like antibiotic treatment [114], may induce fast endotoxin release due to cell lysis. In part, such a drawback, along with phage regulatory gaps and incompliances, have contributed to halt phage therapy approval for human use [115]. However, emerging initiatives, such as the phage therapy framework initiative based on magistral preparations that Belgium has recently undertaken [116], may help to tackle and overcome the above-mentioned issues and underpin an expedited transfer to the clinic.

#### 3.3.3. Probiotics and Prebiotics

The majority of the current microbiota-based therapeutics target the modulation of intestinal microbial composition through exogenous administration of live microbes, termed probiotics. Probiotics, by definition, are expected to benefit human health and their market has expanded tremendously over the last decades. However, hostile mobile genetic elements have been frequently documented to transfer to and from probiotics [117]. In this regard, antibiotic-resistant genes have been detected in various dietary supplements [118]. Moreover, probiotics have been found capable of secreting an enzyme, identified as a potential etiologic factor in celiac disease [119,120,121,122], and partially failed to produce a significant change on the GM population [123,124,125,126]. In addition, probiotic effects decrease significantly with time in mice [126] and their function markedly declines in products upon storage [127]. Alternative to probiotics, prebiotics are compounds capable of influencing microbiome composition or function in a non-specific and beneficial way. A combination of both, namely synbiotics, is being investigated with as of yet insufficient evidence and unclear benefits [128,129].

Lately, a number of systematic reviews, a meta-analysis, and expert opinions have expressed criticism on the probiotics and prebiotics claimed effects and safety [130,131,132,133,134,135,136].

## 4. Postbiotics: The Changing Paradigm

A new frontier is emerging as a result of a deeper understanding of the role of microbiota derived metabolites, termed postbiotics, in human diseases, which may enable novel therapeutic approaches [137,138].

Upon responding to nutritional cues within the host microenvironment, bacteria generate a large number of metabolites, many of which have undefined biological functions. The metabolites produced by one class of bacteria can influence antibiotic susceptibility of the neighboring bacteria within a niche. Progresses in high-throughput sequencing and metabolomics have promoted the identification of a series of postbiotic compounds that can contribute to directly and specifically manipulate the microbiota and host functions [139]. Many of these postbiotics derive from the transformation of specific dietary components by specialized microbe species expressing the required enzymes [140,141].

Noteworthy, the large variability of the GM composition among individuals reflects on its metabolic and functional phenotype, which explains the large array of microbial-derived metabolites among the population [142].

Metabolomic studies have stressed that the microbial metabolite population remarkably differs between diseased and healthy subjects [143,144]. The disruption of the host-microbiota balance leads to a number of intestinal and extra-intestinal diseases. Gao et al. discovered that mice fed with butyrate-enriched high-fat diet have increased thermogenesis and energy expenditure and were less prone to obesity than control mice [145]. Moreover, Rouse et al. [146] demonstrated that pre-treatment with dietary indole derivatives, such as 3-indolelactic acid (3IA) and diindolylmethane, prevents the development of experimental autoimmune encephalomyelitis. On the other hand, Koeth et al. showed that the microbial metabolism of dietary L-carnitine produces trimethylamine-N-oxide and accelerates atherosclerosis in mice [147].

Therefore, evidence is continuously emerging on the importance of such molecules that can play a bridge role between the host and the microbe. Potentially, these natural compounds may be exploited to generate a new class of therapeutics not aiming at the elimination of the invading microorganisms, but rather at manipulating the crosstalk among bacteria and the host to restore a healthy balance.

Postbiotics are physiologically abundant at variable concentrations with low toxicity concerns, showing defined chemical structures, safety dose parameters, and long shelf life (up to 5 years as dietary supplements) [148,149], features that make them highly therapeutically attractive. Moreover, Shenderov and co-workers [150] have reported that these compounds show suitable features for absorption, synthesis, transmission, and excretion, which could suggest a high capacity to signal various organs and tissues in the host, resulting in several biological responses.

In general, postbiotics have been recognized as being responsible for biologically relevant local and systemic effects dependent on the qualitative and quantitative composition of the GM. Several of these metabolites are found in many extra-intestinal tissues and impact significantly on the intestinal immune homeostasis, vascular function, neurological behavior, and energy metabolism [151,152,153] (Table 3).

### 4.1. Major Postbiotic Groups

Postbiotics can be classified by their chemical nature, such as lipids, carbohydrates, proteins, organic acids, vitamins/co-factors, peptidoglycan-derived muropeptides, or lipoteichoic acids, or by their physiological functions, including immunomodulation, anti-inflammatory, hypocholesterolemic, anti-obesogenic, anti-hypertensive, anti-proliferative, and antioxidant activities [149,181] (Table 4).

The main postbiotics groups are:

1. Carbohydrate metabolites—anaerobic bacteria produce short-chain fatty acids (SCFAs) through carbohydrate fermentation in the intestine. They are formed starting from polysaccharide, oligosaccharide, protein, peptide, and glycoprotein precursors [193]. In particular, bacteria of the *Bacteroidetes* phylum are good producers of acetate and propionate SCFAs, whereas those in the Firmicutes phylum are efficient butyrate producers [194].
2. Amino acid and related metabolites—proteins are metabolized by many bacterial species, such as *Bacillus, Clostridium*, *Streptococcus*, *Lactobacillus*, and *Proteobacteria* phyla.
3. Lipid and bile acid metabolites—the GM alters bile acids through various modifications [195]. More than 20 different secondary bile acids are generated, including deoxycholic acid and lithocholic acid, as well as phosphatidylcholine is metabolized to produce trimethylamine-N-oxide.

Several host cell receptors can sense microbial metabolites: (i) purinergic receptors, such as P2X_7_, detect microbial and host-derived nucleotides [196], (ii) the membrane bile acid receptor and Farnesoid X receptor bile acids and xenobiotic metabolites [197,198], (iii) G-protein-coupled receptors (GPRs), such as GPR43 and GPR41, SCFAs, and (iv) the aryl hydrocarbon receptor (AhR) and pregnane X receptor (PXR) tryptophan (Trp), indole, bile acids, and toxicant metabolites [199,200,201] (Table 4).

### 4.2. Targeting of the Host–Microbiota–Pathogen Interface

Some postbiotics seem to play a dual role during bacterial infections by acting on the pathogen while strengthening the host’s resistance to disease and immunopathology. Although the relative mechanisms are not fully understood, postbiotics functional properties encompass anti-inflammatory, immunomodulatory, and antimicrobial that avail microbiota homeostasis and/or host metabolic and signaling pathways [82,83,202]. Here below, the main recognized ways and functions by which postbiotics can exert their potential therapeutic role are briefly discussed.

#### 4.2.1. Signaling Molecules

Postbiotics can mediate the host–microbe communication. Some postbiotics act on downstream signaling pathways of the microbiome, rescuing directly or indirectly the relative dysregulated processes. As such, they have the potential to counteract and correct the negative effects of dysbiosis as well. Through PRRs, such as NLRs (nucleotide-binding oligomerization domain and leucine-rich repeat-containing receptors), the host can recognize microbial elements, which can result into downstream antimicrobial responses or tolerance [203].

The SCFA butyrate has shown a double role in cellular metabolism and as a signaling molecule [86]. SCFAs are agonists for several GPRs on the cell surface, such as GPR43 and GPR41 [204,205], thus activating MAPKs, the extracellular-signal-regulated kinase 1 and 2, c-Jun N-terminal kinase and p38/MAPK [206]. Moreover, being able to translocate inside cells via active transport by the sodium-coupled monocarboxylate transporter 1 [207,208], SCFAs affect transcriptional regulation by inhibiting nuclear class I histone deacetylases (HDACs) and activating histone acetyltransferases. As reported by Steliou et al. [209], butyrate and, to a lesser extent, propionate are known to act as HDAC inhibitors [210,211]. Furthermore, Alex and coworkers [212] attributed to butyrate the ability to act as an agonist for the peroxisome proliferator-activated receptor-γ, with a consequential impact on fatty acid deposition and glucose metabolism. 

#### 4.2.2. Enhancement of Epithelial Barrier Function 

Postbiotics can promote colonization resistance by enhancing barrier function within the epithelial lining. Elamin et al. [213] showed that butyrate exerted a protective effect on the intestinal barrier function in Caco-2 cell monolayers by upregulating the expression of mucin 2 [214], which structurally reinforced the mucous layer and enhanced the protection against luminal pathogens. Levy et al. [178] demonstrated that exogenous polyamines, such as spermine and monoamine histamine, reducing the release of IL-18, an epithelial repair and barrier protective cytokine, can damage the intestinal epithelium by inhibiting the activation of the NLRP6 inflammasome. Taurine, an endogenous bile acid derived microbial metabolite, successfully contrasted the negative spermine and histamine effects, indicating a finely tuned balance of opposing bacterial stimuli [178]. Furthermore, Assa et al. [215] investigated the impact of vitamin D deficiency on the infection-related perturbation of the intestinal epithelial barrier in vitro and on *Citrobacter rodentium*-induced colitis in mice, a pathological condition that causes increased colonic permeability to macromolecules [216]. Vitamin D deficiency further increased intestinal paracellular permeability, supporting the vitamin D role in maintaining the epithelial barrier mucosal integrity.

#### 4.2.3. Immunomodulatory Activity 

Postbiotics have been found to stimulate anti-inflammatory immune responses and act as immunomodulators in various animal models [148].

Potential metabolite therapeutics include SCFAs, owing to a demonstrated anti-inflammatory activity and an observed alteration in certain diseases, such as IBD [163,217,218]. Maciejewska et al. revealed altered butyrate levels in high fat diet models [219,220]. Butyrate promotes the differentiation of the colonic and small intestine Tregs, crucial for intestinal homeostasis, through GPRs, including GPR41, GPR43, and GPR109A. GPRs are known for being involved in NLRP3 inflammasome activation as well [101]. Venegas and coworkers [221] showed that SCFAs exert anti-inflammatory effects on the intestinal mucosa by HDACs inhibition and by activating the GPRs present in the intestinal epithelial cells and immune cells. Moreover, Cox et al. [222] demonstrated that SCFAs were capable of inducing prostaglandin E2 release and the expression of IL-10 through pertussis toxin-sensitive GPRs, thereby inhibiting inflammatory responses in human monocytes. Additionally, flavonoids have been implicated in therapies for metabolic diseases [223] owing to their ability to interfere with inflammatory signaling [224]. In a mouse model of metabolic syndrome, Rivera et al. [225] showed as the administration of quercetin increased plasma concentration of adiponectin, reduced the tumor necrosis factor-α secretion and the expression of the proinflammatory inducible nitric oxide synthase in visceral adipose tissue. Son et al. [226] observed that taurine reduced inflammation in rat models of IBD, thus decreasing colonic damage and the incidence of diarrhea. Additionally, *Lactobacilli* derived postbiotics have been found to reduce tissue inflammation by modulating the inflammatory mediator secretion and nuclear factor-κB (NF-κB) activation [227,228,229,230]. On the other hand, recent works have shown that higher trimethylamine-N-oxide levels induced the activation of the NF-κB pathway and increased the expression of pro-inflammatory cytokines, adhesion molecules, and chemokines [191,231].

#### 4.2.4. Antimicrobial Activity 

Several known and unknown postbiotic compounds, such as bacteriocins, enzymes, small molecules, and organic acids, exhibit bacteriostatic or bactericidal properties against both Gram-positive and Gram-negative microorganisms [232]. For instance, nisin A, produced by *Lactococcus lactis*, is a pore-forming bacteriocin that binds the peptidoglycan precursor lipid II preventing its binding with penicillin and leading to rapid cell death [233]. Synergism between bacteriocins and commensals like *L. acidophilus* has been reported and this combined action was able to inhibit the growth of *Salmonella enterica*, *S. aureus,* and *Bacillus cereus* [233]. Vilchèze et al. [234] revealed the ability of vitamin C to impair bacterial biofilm formation by inhibiting the production of extracellular polymeric substances.

#### 4.2.5. Antiproliferative Activity 

Not surprisingly, the host–GM axis may affect cancer genesis and development as well. Postbiotics have also shown antiproliferative activity against colon cancer cells, likely by controlling immune responses and through the activation of pro-apoptotic cell death pathways [235,236]. In particular, butyrate and propionate inhibited host’s tumor cell HDACs by a mechanism that is still obscure. Flavonoids, such as quercetin, suppressed tumor growth by inhibiting protein tyrosine kinases in colon cancer cells [237]. Anthony et al. [238] reported anti-proliferative apoptotic effects of indole-3-carbinol (I3C), produced by Trp metabolism, on human breast cancer cells. I3C showed an anti-proliferative response by selectively stimulating the interactions between the stem/progenitor cell marker nucleostemin with murine double mutant 2, which triggered the p53 apoptotic response. Furthermore, Sreenivasa and coworkers [239] revealed the I3C ability to inhibit cell growth and induce apoptosis in prostate cancer cells by inhibiting the NF-κB activation.

The many implications of the above-mentioned properties and ways of action of postbiotics infer their potential pivotal role as drivers for the development of personalized therapies aiming to manipulate the host microbiota [137]. The following section describes the nature and known mechanisms of the most promising compounds so far identified, focusing specifically on indoles.

## 5. Tryptophan-Derived Postbiotics: Indoles

The complex host–microbiota crosstalk is modulated by a large array of metabolites. Among all, Trp metabolites take a center stage. 

Trp is one of the nine essential amino acids supplied by diet and its metabolic pathways generate a number of key molecular modulators of the GM [240,241]. Such metabolites are of both host and microbial origins. The 99% of diet Trp is metabolized along three major pathways in the gastrointestinal tract: (i) via specific bacterial strains into indole and indole derivatives (microbial origin) [143,144], (ii) via the kynurenine pathway in both immune and epithelial cells by indoleamine 2,3-dioxygenase 1 (host origin) [242], and (iii) via the serotonin production pathway in enterochromaffin cells by Trp hydroxylase 1 (host origin) [243].

The enzyme tryptophanase, expressed in many Gram-negative and Gram-positive bacterial species including *E. coli*, *Clostridium* spp., and *Bacteroides* spp. leads indole formation [151,244,245,246,247]. *Clostridium* spp. convert Trp into tryptamine, 3-indolelactic acid (ILA) and indole propionic acid [143,248,249]. Likewise, *Peptostreptococcus* spp. are known to convert Trp in indole acrylic acid and indole propionic acid. *Lactobacillus* spp. convert Trp to 3-indole-carboxaldehyde (3-ICA) and ILA via the aromatic amino acid aminotransferase and an indoleacetic acid dehydrogenase [179,250]. Several *Bacteroides* species have been reported to produce ILA and indoleacetic acid [251], whereas *Bifidobacterium* spp. to produce ILA [251,252].

Indoles are a major class of postbiotics that has gained considerable credit owing to their capacity to extend health span across a broad range of evolutionarily diverse species from different phyla [253]. The indole and indole derivatives synthesized in the gastrointestinal tract act as a possible link to microbiota dysbiosis by affecting host immune reactivity, epithelial barrier function, and pathogen colonization [254]. These compounds seem to function through a what could be defined as a two-faced Janus’s mode of action across the intertwining liaisons connecting the host and microbes as explained below. 

### 5.1. Indoles at the Microbiota–Pathogen Interface-Indoles as Quorum Sensing Signals

The appraisal of indole as a quorum sensing (QS) molecule is of relatively recent origin [245]. As an intercellular signaling molecule, indole controls spore formation, plasmid stability, drug resistance, and biofilm formation [255,256,257,258,259]. Recent works have unraveled the indole role in the acquisition of antibiotic resistance through the formation of antibiotic-tolerant persister cells through probable oxidative stress and phage shock pathways [259], which supports the hypothesis that indole is involved in antibiotic resistance processes via multidrug transport [260,261,262]. Chimerel et al. [263] showed that indole could act as a proton ionophore and inhibit cell division by reducing the electrochemical potential when indole crosses the membrane. Kim et al. [264] demonstrated that indole caused toxicity to *P. putida* and interfered with protein folding. Highly expressed genes coding for proteases, molecular chaperones, and tricarboxylic acid cycle enzymes can play crucial roles in indole-induced stress conditions. Indole has been reported to act as an antivirulence compound against *E. coli*, *P. aeruginosa,* and *S. aureus* [255,256,257,265]. Lee et al. [256] demonstrated that indole modulates the expression of virulence and regulatory genes, previously reported as being substrate for the *P. aeruginosa* QS system.

### 5.2. Indole at the Host–Microbiota Interface-Indoles as an Intercellular Signal in Microbial Communities

The functional effects of certain indoles can be sorted into two categories: (i) integration into host intracellular metabolism and (ii) receptor-mediated metabolite sensing. In the first case, the microbiome supports tissues and organs as an endocrine energy source [266]. In the second case, host’s eukaryotic cells recognize microbiome-derived metabolites, which trigger receptor-mediated signaling cascades and cell-specific transcriptional responses either locally or systemically. As reported above for multiple postbiotics, such signaling functions and their interaction with the immune system have been found to particularly characterize indole derivatives [267]. The major recognized indole and indole derivative functions are concisely summarized hereinafter. 

#### Indoles as Ligands of the Aryl Hydrocarbon Receptor

Recent works on indoles generated via microbial metabolism show that several host receptors, which include the AhR and PXR, could be important targets for regulating the host–microbe homeostasis [201,268]. In this section, we focused on the AhR, since its promiscuous ligand binding site is capable of accommodating diverse small bacterial indole metabolites. AhR can be activated by a large number of exogenous-endogenous ligands and, by inducing gene expression, AhR regulates many physiopathological events, such as cell differentiation, proliferation, adhesion, apoptosis, and migration [268,269]. This evidence has sparked new understanding of the AhR signaling pathways and its interaction with endogenous metabolites. It is clear that AhR has multiple functions beyond toxicology, to include developmental biology, and bidirectional communication with the microbiome for tuning host immunity, tolerance, and metabolism [268,270] (Table 5).

### 5.3. Regulation of the Immune Response

AhR is a transcription factor, highly expressed in immune and epithelial cells [275], that serves as a sensing and modulator system for environmental toxins [276]. This response is mediated by innate and adaptive immune cells through alteration of the CD4^+^ T cell polarization into Tregs or Th17 cells. For instance, 6-formylindolo[3,2-b]carbazole, an endogenous Trp metabolite, promotes, via AhR activation, the generation of Th17 cells [277]. Likewise, kynurenine, another Trp metabolite, promotes pTreg generation [278]. Moreover, AhR activation by the xenobiotic ligand 2,3,7,8-tetrachorodibenzodioxin enhances the generation of Tregs from CD4^+^ cells in vitro and inhibits the production of Th1 and Th17 cells [279,280]. AhR promotes Th17 differentiation through different mechanisms, including direct binding to the *Il17* gene locus or the inhibition of the signal transducer and activator of transcription 1 phosphorylation, which knocks down Th1 in favor of Th17 differentiation [280,281]. Such a regulatory role on the T-cell compartment underpins the AhR potential as a therapeutic target for the treatment of autoimmune disorders [282,283,284,285,286].

### 5.4. Regulation of Intestinal Homeostasis

AhR plays multiple functions, which include antimicrobial defense, tissue protection and repair, and toxin clearance. In fact, AhR activation promotes the modulation of antimicrobial activity via Th17 cell activation [270], increased epithelial barrier function, epithelial cell repair [287], IL-22 mediated protection [179], and control of inflammation via Treg activation [278]. Furthermore, AhR upregulates the cytochrome P450 enzymes enhancing the metabolism of harmful toxicants.

AhR deficiency increased the severity of T cell or sodium dextran sulfate (SDS) induced experimental colitis in mice. In the same models, Zelante et al. [179] associated AhR deficiency related colitis to an altered production of IL-22. Lamas et al. [288] also demonstrated, in mice deficient in the caspase recruitment domain (Card) 9, an IBD susceptibility gene, that the impaired capacity of the dysbiotic GM to catalyze Trp conversion into AhR ligands reflected into lower IL-22 levels and a higher Card9^−/−^ mice susceptibility to SDS-induced colitis. Some evidence exists in humans as well. Monteleone et al. [289] reported as the pharmacological activation of AhR decreases the proinflammatory cytokine IFNγ and increases the production of IL-22 in lamina propria mononuclear cells from IBD patients.

The above evidence clearly suggests that the development of suitable postbiotic delivery strategies may greatly benefit the treatment of autoimmune inflammatory and neoplastic diseases as well as antibiotic resistance.

## 6. Nonantibiotic Indoles as Novel Therapeutic Tools

The capacity of certain indoles to act across the host–microbiota–pathogen axis evokes new scenarios in infectious disease treatment and management. Intriguingly, such indoles may enable novel nonantibiotic therapies where killing the microbes is not the primary endpoint. Therefore, by avoiding selective pressure on the pathogen, microbial resistance could be prevented, while likely allowing treatment of multidrug resistant strains.

Preclinical evidence suggests that indole postbiotic-based treatments can either moderate the inflammatory response to infection or limit microbial growth by blocking access to host resources, without exerting toxic antimicrobial effects [6,290]. Therefore, modulating bacterial processes not fundamental for cell survival is not supposed to trigger the development of defensive mechanisms. Moreover, a better understanding, at a molecular level, of processes, such as QS, could enable manipulation of the biological signatures allowing prevention or inhibition of the pathogen ability to establish infection.

In fact, pathogens, to colonize the host, must activate QS signaling to proliferate, form biofilms, and produce virulence factors, suggesting that breaking down this bacterial communication by anti-QS agents could make pathogens more susceptible to host immune responses and antibiotics. In this regard, combination therapies coupling an antibiotic with an anti-QS agent are currently considered as one of the most effective clinical strategies for the treatment of bacterial diseases [291,292].

### 6.1. The Case of 3-Indole-Carboxaldehyde (3-ICA)

A postbiotic of particular interest is 3-ICA, a Trp metabolite that seems to exert dual beneficial effects on the organism as well as the GM. 3-ICA is abundantly produced by *L. reuteri* in the gut, via aromatic amino acid aminotransferase, and acts as a ligand of AhR [179]. Considering the previously discussed activity of AhR in protecting from inflammatory damage and maintaining barrier integrity [131], it is not surprising that a diet rich in AhR ligands can prevent or halt tumorigenesis [293] and that restoring AhR signaling rescues metabolic syndrome [294], a disorder characterized by a cluster of diseases, including visceral obesity, low high-density lipoprotein, hypertension, hyperglycemia, and hypercholesterolemia [295]. This rationale has provided the base for the development of 3-ICA delivery approaches aimed at the treatment of dysreactive immune disorders and infections [296,297].

Overall, 3-ICA can exert an important role at the host–microbiota–pathogen interface. We briefly summarize some published and unpublished results below.

#### 6.1.1. 3-ICA Enhances Epithelial Barrier Integrity

3-ICA is capable of activating ILC3 for IL-22 production via AhR. Sensed by intestinal epithelial cells, IL-22 enhances the epithelial barrier integrity as well as the production of antimicrobial peptides, thus exerting a control over the local microbial load and composition.

Coherently with such premises, 3-ICA administration enhanced the barrier integrity and the production of antimicrobial peptides in murine models of colitis, gastrointestinal, and vaginal candidiasis [78,179]. Thus, the microbe driven IL-22 production [179,298] can untwine how antibiotic-related dysbiosis and cancer therapy may predispose to secondary fungal infections and suggests that therapies aimed at restoring the AhR/IL-22 axis at the mucosal surface could be of therapeutic benefit. To the best of our knowledge, no current or in-development treatments are designed to modulate disease symptoms through epithelial repair.

#### 6.1.2. 3-ICA Reduces Intestinal Inflammation

In a graft-versus-host-disease murine model, the treatment with 3-ICA decreased intestinal epithelial damage, transepithelial bacterial translocation, and inflammatory cytokine production. 3-ICA treatment also led to recipient-strain-specific tolerance of engrafted T cells and upregulated the genes associated with the type I IFN response known to protect against radiation-induced intestinal damage. Thus, 3-ICA, by acting through different downstream effector pathways, may limit intestinal inflammation and damage and may provide a therapeutic option for patients at risk for metabolic syndrome, inflammatory bowel diseases, and graft-versus-host-disease [299].

#### 6.1.3. 3-ICA Attenuates Inflammation in Patients with Atopic Dermatitis

A recent work reported that 3-ICA levels on the skin surface are significantly lower in patients with AD [300]. Topical application of 3-ICA alleviated skin inflammation in a mouse model of AD-like dermatitis, in part through the inhibition of thymic stromal lymphopoietin expression in keratinocytes in an AhR dependent fashion.

#### 6.1.4. 3-ICA Acts as a QS Signaling

Interestingly, 3-ICA has also been shown to regulate virulence of pathogenic bacteria by interfering with bacterial small-molecule signaling [301]. This finding suggests that the control of the microbial composition and fitness could be an additional mechanism through which 3-ICA may exert its therapeutic efficacy at the host/microbe interface.

**Figure 1:**
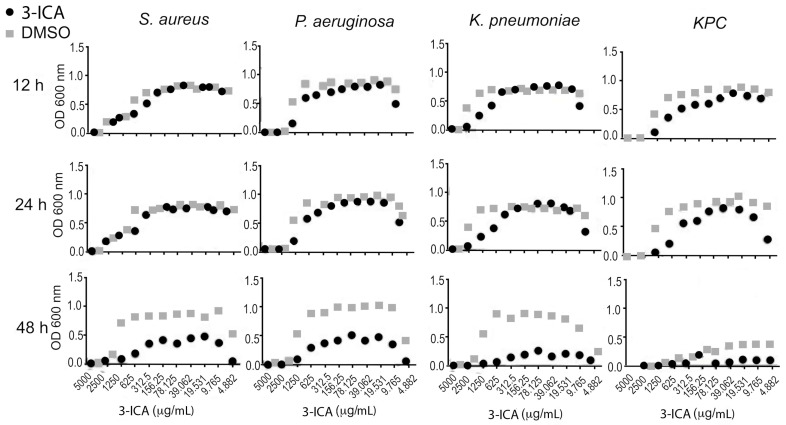
3-ICA antimicrobial activity in vitro. Bacteria are inoculated into a liquid growth medium in 96-well microtiter plate format in the presence of different concentrations of 3-ICA to determine the minimum inhibitory concentration (unpublished data, see Supplementary Materials).

In an in vitro preliminary study on bacterial growth, 3-ICA exhibited potent antimicrobial activity against *S. aureus*, *P. aeruginosa*, *K. pneumoniae,* and, of great potential interest, against *K. pneumoniae* carbapenemase (Figure 1, Supplementary Materials). Preliminary as they are, these results suggest that the beneficial effects of 3-ICA may encompass an activity upon the local microbiota composition.

#### 6.1.5. 3-ICA Ameliorates Respiratory Allergic Bronchopulmonary Aspergillosis

**Figure 2:**
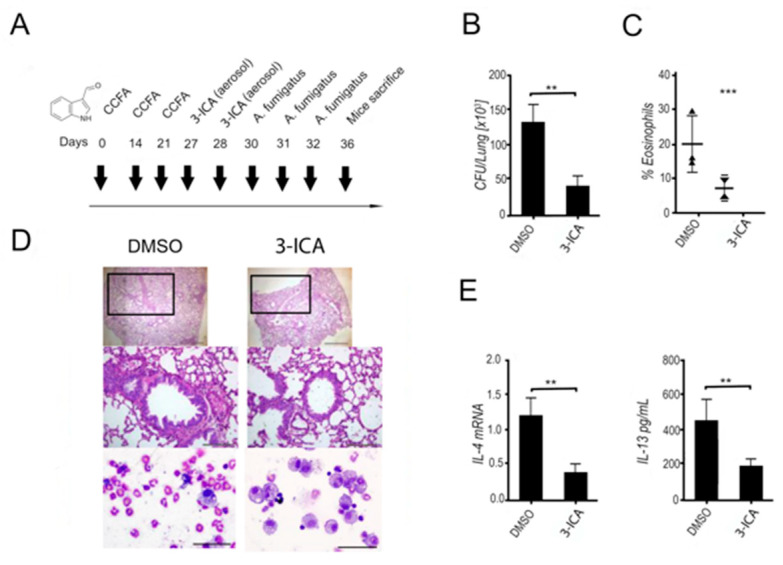
3-ICA decreases lung immune pathology in ABPA. (**A**) ABPA mouse model. (**B**) Fungal growth (CFU, mean ± SD), (**C**) eosinophil recruitment (%), and (**D**) periodic acid Shiff staining of the lungs of C57BL/6 mice with ABPA and treated with 3-ICA (at 7 days post elicitation) or the vehicle DMSO. (**E**) *Il4* and *Il33* specific transcripts in the lungs of ABPA mice treated with 3-ICA. (*** *p* < 0.001; ** *p* < 0.001; unpublished data, see Supplementary Materials).

Recent unpublished research showed that allergic bronchopulmonary aspergillosis (ABPA) could be successfully treated by 3-ICA administration. In fact, 3-ICA intranasal delivery decreased lung immune pathology in ABPA (Figure 2, Supplementary Materials). In particular, 3-ICA administration reduced fungal colonization and eosinophil numbers, mucin production, inflammatory cell influx, and peribronchial fibrosis. Moreover, 3-ICA reduced the *Il4* and *Il13* gene expression in the lungs, underlining the improvement of the local allergic response to the fungus.

The above multiple 3-ICA observed activities, along with the growing knowledge on the AhR importance in restoring microbial eubiosis, immune tolerance, and resistance to pathogens [179,302], underpin a future major role of postbiotics to treat immune and autoimmune diseases as well as bacterial infections [303].

The above findings encourage the development of new nonantibiotic treatments for infectious diseases with a likely tremendous impact on the prevention of AMR. Although as yet to be fully disclosed, even in light of the absence of toxic events, 3-ICA could be proposed as a valid therapeutic alternative for difficult to treat infections.

## 7. Conclusions

On the basis of a metabolic profiling approach, the concept of pharmaco-metabonomic, proposed by Clayton et al. [304], suggests how mining the microbiota may lead to personalized treatment, in which drug-induced responses in individuals can be predicted from a metabolic signature. Indeed, the growing knowledge on the diet–microbiota–host interface fosters opportunities for new therapeutic approaches, based on the selective alteration of microbial metabolite production to support human health and prevent disease. Not only the GM itself may be a largely untapped source of new drugs or lead therapeutic molecules, but also continuous advances will help building novel platforms for the identification and development of new therapeutic strategies for chronic diseases centered on a dual targeting at the host–microbe interface [305,306]. At the same time, detection of microbial metabolites as new sensors will provide new insights into the mechanisms by which the host determines the behavior of the microbial environment, with direct effects on starting inflammation versus keeping homeostasis intact. Moreover, the identification and comprehension of postbiotic mechanisms of action may promote a lead discovery phase of breakthrough therapeutics that centers on correcting the dysfunctional host–pathogen interaction and go beyond the classical mechanisms targeting either the microbes or the host.

Albeit in its infancy, the nonantibiotic strategy to infections shows multiple advantages and may represent a new frontier market for the pharmaceutical industry. The data and considerations reported in this review, although a drop in the sea of knowledge, clearly prove the great potential of this new strategy. What should be minded is that aggressive approaches to infection are tremendously biased, as we are today paying an expensive tribute to decennial mismanagement and careless use of antibiotics. To win our battle against the serious complications of AMR, we need to cope with the overwhelming pathogen capacity to adapt and to develop a very effective defensive arsenal. Therefore, we need to develop the capacity to communicate with the microbes in order to restore a peaceful coexistence that will naturally evolve towards a healthy ecosystem, making eradication unnecessary. Eventually, this new philosophy will surely pay off as it will allow one to prevent the many pathologies associated, not only to infections, but also to the side effects due to the excessive and reckless use of antibiotics. These new opportunities are directly connected to the observed double nature of postbiotics, which act around the host–microbe boundaries. Among all, indoles, in particular 3-ICA, can have a primary role due to their origin and signatures. These compounds possess commercial potential, as they are relatively inexpensive, widely available, and apparently low toxic enabling fast translation to the clinic. In addition, they can be formulated using industrially established technologies. Needless to say, considerable effort is still required in order to fully prove the clinical relevance of most of these compounds. In fact, most studies describing molecular mechanisms of postbiotics have been often performed in vitro or in preclinical settings and their benefits in humans have not been fully elucidated.

Therefore, for the time being, it is not possible to conceive a complete replacement of antibiotic therapies, however a supporting role of postbiotics could be envisaged to improve prevention and control of AMR.

After the first breakthrough discoveries of salvarsan, penicillins, and prontosil antibiotics during the first decades of 1900, due to HDTs and postbiotics, we may now face a second revolution, though requiring a paradigm change in the scientists’ way of perceiving infections. Although as yet the end of the antibiotic era cannot be conceived, the time when acute or chronic bacterial infections used to be treated with “antibiotic-only” therapies may have come to a blunt end.

## Figures and Tables

**Table 1:** Examples of antimicrobial host directed therapies (HDTs) and mechanisms of action (not intended to be exhaustive).

Pathogen	HDTs		Mechanisms of Action	Ref
*Mycobacterium tuberculosis*	Repurposed drug	Imatinib, verapamil, metformin, ibuprofen	Modulation of inflammation and activation of intracellular antimicrobial defenses	[29,37,38,39,40,41,42]
	Cytokine therapy	IL-2, GM-CSF, INF-γ, IL-12	Induction of pro-inflammatory cell signaling	[33]
	Monoclonal antibody	Anti-TNFα, anti-IL-6, anti-VEGF	Reduction of tissue-destructive inflammation by cytokine neutralization	[34]
	Monoclonal antibody	Anti-PD-1, anti-LAG3, anti-CTLA-4	Activation and mobilization of antigen-specific T cells by immune checkpoint inhibition	[35,44]
	Vitamin	Vitamin D3	Activation and augmentation of intracellular antimicrobial defenses (via IFN-γ and IL-15 signaling)	[41,42]
	Cellular therapy	Autologous mesenchymal stromal cells, T cells	Neutralization of tissue-destructive inflammation, enhancement of organ repair, and potentiation of antigen-specific immune responses	[36]
*Streptococcus pneumoniae*	Repurposed drugs	Prednisone	Reduction of tissue-destructive inflammation by activating the glucocorticoid pathway	[44]
		Ibuprofen, statins, indometacin, aspirin	Reduction of tissue-destructive inflammation by inhibiting prostaglandin release via cyclooxygenase inhibition, regulation of MHC molecules	[45,46]
		Glibenclamide	An oral hypoglycemic agent that modulates voltage-gated calcium channels, leading to immunomodulatory effects	[50]
*Bordetella pertussis*	Repurposed drug	Fingolimod	Activates the sphingosine-1-phosphate pathway to improve antigen-specific lymphocyte responses, as well as reduced hyper-inflammation	[51]
	Monoclonal antibody	Antipertussis toxin antibodies	Reduces toxin load via infusion of intravenous immunoglobulins	[47,48,49]

**Table 2:** Examples of microbiota-based therapeutic strategies and development phase (not intended to be exhaustive).

Microbiota Based Therapies		Symptomatology		Trials	Reference
Bacteriophages	Muddy, BPs33ΔHTH-HRM10, and ZoeJΔ45	Cystic fibrosis	*Mycobacterial infection*	Clinical case study	[90]
	OMKO1	Prosthetic vascular graft infections	*Pseudomonas aeruginosa*	Preclinical	[91]
	PP1131 cocktail	Burn wounds infected	*Pseudomonas aeruginosa*	Phase I-II	[92]
	Myoviridae	Enteric infection	*Escherichia coli*	Preclinical	[93]
	phage cocktail	Burn wound infections	*Klebsiella pneumoniae*	Preclinical	[94]
FMT		*Clostridium difficile*		Preclinical	[95]
		Ulcerative colitis		Phase II	[96]
				Phase II	[97]
Probiotics	*Lactobacillus acidophilus* *Saccharomyces boulardii Lactococcus lactis, Lactobacillus rhamnosus, Lactobacillus plantarum, Lactobacillus casei,* *Lactobacillus reuteri, Lactobacillus plantarum,* *Bifidobacterium infantis*	Traveler’s diarrhea, Antibiotic-associated diarrhea, Ulcerative colitis Crohn’s disease, Atopic dermatitis, *Clostridium difficile* Irritable bowel syndrome		Phase II	[98,99,100,101]

**Table 3:** Main postbiotic classes and their biological activities (adapted form 254).

Metabolite	Activities	Reference
Butyrate, acetate, propionate	Preserve mucosal immunity Enhance the regulatory function of Tregs in the large intestine Butyrate suppresses proliferation by acting as a HDAC inhibitor Enhance the protection against infections Activate GPR43 and GPR109a on intestinal epithelial cells, result in the activation of the NLRP3 inflammasome leading to production of IL-18. NF-kB inactivation and suppression of pro-inflammatory cytokines and nitric oxide in neutrophils and mononuclear cells through HDAC inhibition	[154,155,156,157,158,159,160,161,162,163,164,165,166,167,168]
Niacin	Induces anti-inflammatory properties in dendritic cells and macrophages in a GPR109a-dependent manner and suppresses colonic inflammation	[160]
Retinoic acid	Dendritic cell induction of gut-lymphocytes Supports the development of Tregs through TGF-β and suppresses the development of TH17 cells during inflammation, RA is required for the induction of a proinflammatory CD4+ helper T cell response	[169,170,171,172]
Polysaccharide A (PSA)	Suppresses the production of pro-inflammatory IL-17 and promotes expression of IL-10 by CD4+ T cells	[156,173,174]
Bile acids	Regulation of bacterial growth Inhibit the induction of pro-inflammatory genes through NF-kB	[175,176,177]
Taurine	Nlrp6 inflammasome activation and contribution to intestinal homeostasis	[178]
Indoles	Induce IL-22 secretion by ILCs, further driving the secretion of antimicrobial peptides and protection from infections by pathogens Epithelial barrier enhancement	[56,179,180]

**Table 4:** Microbial derived metabolites and their biological targets.

Source	Bacteria	Microbial Metabolite	Family	Receptor	Reference
Fermentation of fibers/carbohydrate metabolism	*Bacteroidetes,* *Firmicutes*	Propionate, Acetate and Butyrate	SCFA	GPR43 GPR41	[182]
Dietary Tryptophan (microbial origin)	*Firmicutes, Lactobacillus, Clostridium, Bacteroides*	5-hydroxy-tryptophan Tryptamine Indoleacetic acid 3-methylindole (Skatole) 3-indole carboxaldehyde Indole-3-sulfate Indole propionic acid 3-indolelactic acid	Indole and Indole derivatives	AhR, PXR	[179,183,184]
Bile acids	*Bacteroides, Bifidobacterium, Clostridium, Lactobacillus, Eubacterium*	Deoxycholic and lithocholic acid	Secondary bile acids	GPBAR1, FXR	[185,186]
Nitrogenous compound	*Escherichia Coli, Enterococcus faecalis, Bifidium bacterium* and *Bacteroides*	Putrescine, Spermidine, spermine	Polyamines	PPARγ, TRPV1	[187,188,189]
Dietary choline	*Actinobacteria* *Bacteroidetes Firmicutes* *Proteobacteria*	Trimethylamine-N-oxide	Amine oxides	FXR	[190,191]
Ginseng	*Bacteroides*	Compound K	Ginsenosides	GPBAR1	[124,192]

**Table 5:** Microbiota and host metabolites with AhR ligand properties (adapted from [271]).

Compound	Origin
Indole Metabolites [268,272]	
Indole	Dietary metabolite and microbiota metabolism
Indolo[3,2-b] carbazole	Dietary metabolite
2-(Indol-3-ylmethyl)-3,3′-diindolylmethane	Dietary metabolite
3,3′-Diindolylmethane	Dietary metabolite
Tryptophan Metabolites [268]	
Kynurenine	Host Metabolism
Kynyrenic acid	Host Metabolism
Zanthurenic acid	Host Metabolism
Cinnabarinic acid	Host Metabolism
5-hydroxy-tryptophan	Host Metabolism
Tryptamine	Microbiota metabolism
Indol-3-acetic Acid	Microbiota metabolism
3-methylindole (Skatole)	Microbiota metabolism
3-indole-carboxaldehyde	Microbiota metabolism
Indoxyl-3-sulfate	Microbiota and Host Metabolite
Arachidonic Acid Metabolites [273,274]	
Lipoxin 4A	Host Metabolism
Prostaglandin -PGG2	Host Metabolism
Hydroxyeicosatrienoic acid	Host Metabolism
Heme-derived [275]	
Indigorubin Bilirubin	Host Metabolism
Indigo Biliverdin	Host Metabolism

## Supplementary Materials

The following are available online at https://www.mdpi.com/1999-4923/12/7/624/s1.
