# Supplementary material for: Postbiotic-Enabled Targeting of the Host-Microbiota-Pathogen Interface: Hints of Antibiotic Decline?
Source: Pharmaceutics. 2020 Jul 4;12(7):624. doi: 10.3390/pharmaceutics12070624 (PMC7408102; doi:10.3390/pharmaceutics12070624)
Supplement: Supplementary file 1 [file pharmaceutics-12-00624-s001.pdf]

pharmaceutics

Supplementary Materials: Postbiotic-Enabled Targeting of the Host-Microbiota-Pathogen Interface: Hints of Antibiotic Decline?

Matteo Puccetti, Styliani Xiroudaki, Maurizio Ricci and Stefano Giovagnoli *

Materials and Methods

Mice

C57BL/6 mice, 8–10 weeks old and cystic fibrosis (CF) mice homozygous for the Phe508del-Cftr allele, which had been backcrossed for 12 generations to the C57BL/6 strain (Cftrtm1EUR, Phe508del, abbreviated CftrF508del/F508del), were obtained from B. Scholte (Erasmus Medical Center) were housed in a controlled environment at the Animal Facility of Perugia University and provided with standard rodent chow and water. CF mice were provided with a special food consisting of an equal mixture of SRM-A (Arie Blok, Woerden, The Netherlands) and Teklad 2019 (Harlan Laboratories, San Pietro al Natisone, Udine, Italy) and water acidified to pH 2.0 with HCl and containing 60 g/l PEG 3350, 1.46 g/l NaCl, 0.745 g/l KCl, 1.68 g/l $NaHCO_3$ and 5.68 g/l Na_2SO_4. Newborn mice were genotyped by cutting a small piece of tail 12 days after birth. Male and female mice were used in all studies. Mouse experiments were performed according to Italian Approved Animal Welfare Authorization 360/2015-PR and Legislative Decree 26/2014 regarding the animal license obtained by the Italian Ministry of Health lasting for 5 years (2015–2020).

Histology and immunofluorescence

Organs were removed and fixed in 10% formaldehyde saline. Tissues were embedded in paraffin, and for each mouse, three 5-µm sections are cut, at two levels separated by 100 µm, and stained with periodic acid–Schiff (PAS) or Hematoxylin Eosin (H&E). All histology sections were examined in a blinded fashion by two observers independently. For immunofluorescence, sections were incubated at 4°C with the primary rabbit anti-Ki-67 antibody followed by secondary FITC-labelled goat-anti-rabbit (Abcam). DAPI was used to detect nuclei. Photographs were taken using a high- resolution Olympus DP71 microscope.

RNA extraction and quantitative RT-PCR (qRT-PCR)

Total RNA was extracted from purified cells or different organs by the TRIzol method (Invitrogen, Milan, Italy) according to the manufacturer's protocol. The cDNA synthesis kit (BioRad, Milan, Italy) was used for reverse transcription according to the manufacturer's protocol. A real-time PCR amplification (PCR using CFX96 Touch™ Real-Time PCR Detection System) was performed using SYBR Green QPCR master mix (Agilent Technologies, Milan, Italy) under the following conditions: 45 cycles of 95°C for 1 min, appropriate annealing temperature for 1 min, and 72°C for 30 sec. All reactions were repeated at least three times independently to ensure the reproducibility of the results.

ELISA

Cytokine content was determined by enzyme-linked immunosorbent assays on tissue homogenates. Briefly, ELISA plates were coated with 50 µl of anti-cytokine capture antibody at 4 °C overnight. Plates were then washed × 3 with 0.05% PBS-Tween (PBST) and coated for 1 h with the 150 µl 1% BSA/ PBS blocking buffer. Samples or standards were added in duplicates (50 µl per well) and

incubated 2h at room temperature. Wells were washed × 3 with PBST and incubated with 50 µl of anti-cytokine detection antibody at 4 °C overnight. Wells were then washed × 3 with PBST and incubated with 50 µl of avidin-HRP at room temperature for 30 min. Thereafter, wells were washed × 5 with PBST and incubated with 50 µl per well of a substrate. The reaction was stopped after 15 min with 1 M H_2SO_4 and absorbance was measured using a TECAN microplate fluorescence reader (Infinite M200) at 405 nm and 570 nm. The PCR primers sequences (5′-3′) were as follows (Table S1).

Table S1. PCR primers sequences.

S100a8	TCGTGACAATGCCGTCTGAACTG	TGCTACTCCTTGTGGCTGTCTTTG
S100a9	CGCAGCATAACCACCATCATC	GCCATCAGCATCATACACTCC
Ahr	CACTGGATGCGTAGGTTCTTGG	TCTTCATCCGTCAGTGGTCTC
Il1b	TGACGGACCCCAAAAGATGAAGG	CCACGGGAAAGACACAGGTAGC
Il17a	GACTACCTCAACCGTTCCAC	CCTCCGCATTGACACAGC
Il10	CCCTTTGCTATGGTGTCCTT	TGGTTTCTCTTCCCAAGACC
Nlrp3	TACGGCCGTCTACGTCTTCT	CGCAGATCACACTCCTCAAA
Il1ra	GACCCTGCAAGATGCAAGCC	GAGCGGATGAAGGTAAAGCG
Cyp1a1	GGTTAACCATGACCGGGAACT	TGCCCAAACCAAAGAGAGTGA
Tnf-α	GCCTCTTCTCATTCCTGCTTG	CTGATGAGAGGGAGGCCATT
IFN-γ	AAAGAGATAATCTGGCTCTGCA	GCTCGAGACAATGAACGCT
KC	CGCTGCTGCTGCTGGCCA	GGCTATGACTTCGGTTTGGGTGCAG
Il22	AATCTATGAAGTTGGTGGGA	ACTGACTCCTCGGAACAGTT

In vitro 3-ICA antibacterial activity

Bacterial medium turbidity (McFarland standards, McF) was quantified through VITEK® 2 DensiCHEK™ Plus. All bacterial strains (*K. pneumoniae*, *KPC*, *P. aeruginosa*, *S. aureus*) were inoculated in tryptic soy broth (TSB) medium (Sigma-Aldrich, Milan, Italy) and plated in untreated 96 well plate (flat bottom) to the final concentration of 0.25, 0.5 and 0.75 McF. 1:2 ten scalar dilutions of 3-ICA and DMSO, as vehicle control, were made in TSB, before adding the bacteria. Bacterial growth has been monitored through ELISA microplate reader, measuring the OD 600 nm absorption at different time points (12h, 24h and 48h).

Allergic Bronchopulmonary Aspergillosis (ABPA) and treatment

Mice were anesthetized in a plastic cage by inhalation of 3% isoflurane (Forane Abbot) in oxygen before intranasal instillation. Mice received an i.p. and s.c. injection of 5 µg of cell culture filtrate Ags (CCFA) dissolved in incomplete freund's adjuvant (Sigma-Aldrich, Milan, Italy) followed by two consecutive intranasal injections, two and three weeks apart, of 20 µg CCFA. A week after the last intranasal challenge, mice received 10^7 *A. fumigatus* resting conidia. 3-ICA aerosolized (166 µg/mL, 30 min daily) or PBS as a vehicle control were administered on day +27 and +28, between CCFA sensitization and *A. fumigatus* infection. Mice were sacrificed 6 days post infection. Paraffin-

embedded tissues were stained with PAS and with Masson's trichrome staining to investigate the collagen deposition. Photographs were taken using a high- resolution Olympus DP71 microscope.

Statistical analysis

Student's t-test, one- or two-way ANOVA with Bonferroni post-hoc test was used to determine the statistical significance. Significance was defined as $p < 0.05$. Data are pooled results (mean ± SEM) or representative images from three experiments. GraphPad Prism software 6.01 (GraphPad Software) was used for analysis.
